# What to Do When You Can’t Transfuse: Update on Optimizing Outcomes for Jehovah’s Witness Patients

**DOI:** 10.3390/hematolrep18050069

**Published:** 2026-09-22

**Authors:** Abdenour Abib, Nadia B. Hensley, Linda M. S. Resar, Steven M. Frank

**Affiliations:** 1 Department of Anesthesiology/Critical Care Medicine, The Johns Hopkins Medical Institutions, Baltimore, MD 21287, USA; aabib1@jh.edu (A.A.); nhensle2@jhmi.edu (N.B.H.); 2 Center for Bloodless Medicine and Surgery, Department of Medicine (Hematology), Oncology & Institute for Cellular Engineering, The Johns Hopkins Medical Institutions, Baltimore, MD 21287, USA; lresar@jhmi.edu; 3 Center for Bloodless Medicine and Surgery, Department of Anesthesiology/Critical Care Medicine, The Johns Hopkins Medical Institutions, Baltimore, MD 21287, USA

**Keywords:** Jehovah’s Witness, bloodless medicine, patient blood management, transfusion, preoperative anemia, cell salvage, autologous donation

## Abstract

Patients who decline allogeneic blood transfusion for religious reasons, most commonly Jehovah’s Witnesses, represent a growing population requiring specialized perioperative and inpatient care. Over the past decade, comprehensive “bloodless medicine” programs have emerged to safely manage these patients using principles of patient blood management, including preoperative anemia diagnosis and treatment with oral or intravenous iron and erythropoiesis-stimulating agents, intraoperative blood conservation techniques such as cell salvage, acute normovolemic hemodilution, antifibrinolytics, controlled hypotension, and meticulous surgical technique, and postoperative strategies to minimize iatrogenic blood loss from phlebotomy. This review synthesizes evidence from a large single-institution experience, encompassing propensity-matched cohort studies of adult, pediatric, and surgical/medical subgroups, alongside novel tools such as a preoperative hemoglobin target algorithm and comparative data on intravenous iron versus red cell transfusion. We also discuss a March 2026 update to Jehovah’s Witness doctrine permitting preoperative autologous blood donation as a matter of personal conscience, and its practical implications for clinical teams. Across these studies, patients receiving bloodless care demonstrated morbidity, mortality, and length of stay comparable to or better than those receiving standard transfusion-inclusive care, with some analyses showing reduced hospital-acquired infection rates and consistently lower hospital costs and charges. A 10-year financial analysis further demonstrated that a comprehensive blood management program generated a greater than sevenfold return on investment while maintaining equivalent clinical outcomes. Collectively, these findings support bloodless medicine as a safe, cost-effective, and increasingly generalizable model of high-value perioperative care.

## 1. Introduction

Blood transfusion remains among the most common procedures performed in hospitalized patients, yet a growing population declines this therapy for religious reasons. Members of the Jehovah’s Witness (JW) faith—an estimated 9.2 million people worldwide, including over 1 million in the United States—do not accept allogeneic blood transfusion (ABT), based on doctrine formally adopted in 1945 [1]. Historically, many hospitals turned these patients away or provided inconsistent care due to unfamiliarity with alternatives to transfusion. This began to change in the 1970s, when Denton Cooley and colleagues published a landmark 20-year series describing more than 500 cardiac surgeries performed on JW patients without transfusion [2], and Cooley is widely credited as a founder of modern bloodless medicine.

The field has since been shaped by a decade of large randomized trials, beginning with the Transfusion Requirements in Critical Care (TRICC) trial in 1999, consistently showing that a restrictive transfusion strategy (Hb 7–8 g/dL) produces outcomes equivalent to or better than a liberal strategy (9–10 g/dL) [3]. Combined with growing recognition of transfusion-related immunomodulation and infectious risk, this evidence gave rise to patient blood management (PBM): a multidisciplinary approach built on diagnosing and treating anemia, minimizing blood loss, and tolerating lower Hb thresholds. Bloodless medicine can be understood as “extreme PBM”—the same principles applied to avoid transfusion altogether rather than simply reduce its use [4].

Over the past decade, our institution has developed both a health system–wide PBM Clinical Community and a dedicated Center for Bloodless Medicine and Surgery, caring for more than 2000 adult and nearly 300 pediatric bloodless inpatients [5]. This experience has generated some of the largest propensity-matched outcome data available for patients who decline transfusion. This review summarizes the evidence-based approach to bloodless care across the preoperative, intraoperative, and postoperative periods; the doctrinal basis for JW blood-refusal practices, including a significant March 2026 update; and outcome and cost-effectiveness data supporting bloodless medicine as a model of high-value care.

Table 1 summarizes the core bloodless medicine techniques applied across the preoperative, intraoperative, and postoperative periods that form the basis of this review, as outlined by Guinn et al. [6].

## 2. What Do Jehovah’s Witness Patients Believe and Why

The JW faith traces its prohibition on blood transfusion to a 1945 doctrinal decision interpreting biblical passages as forbidding the ingestion of blood, including Genesis 9:4, Leviticus 17:10–14, Acts 15:29, and Acts 21:25 [1,6]. Leviticus 17:14 states, “You must not eat the blood of any sort of flesh, because the soul of every sort of flesh is its blood.” JWs interpret these passages as applying to transfusion—a reading that differs from, for example, Jewish or Muslim dietary law, which interprets similar passages as requiring that blood be drained from meat before consumption rather than prohibiting transfusion.

Blood products are traditionally divided into what Jehovah’s Witnesses call “major fractions” (RBCs, white blood cells, platelets, plasma, and whole blood), considered unacceptable by essentially all practicing JWs, and “minor fractions” (albumin, immunoglobulins, cryoprecipitate, individual clotting factors, prothrombin complex concentrate, and hemostatic agents), left to each patient’s personal conscience; more than 90% of JWs accept some or all minor fractions when counseled by a knowledgeable team member [6]. Techniques that keep blood in continuous circuit with the patient’s own circulation—cell salvage, acute normovolemic hemodilution (ANH), cardiopulmonary bypass, hemodialysis, and epidural blood patch—are likewise personal-choice items rather than doctrinal prohibitions, since the blood is regarded as remaining “physically contiguous” with the body [6,7].

Preoperative autologous donation (PAD)—collecting and storing a patient’s own blood in advance for possible reinfusion—was historically forbidden, since it requires blood to be withdrawn and stored outside the body. This changed on March 20, 2026, when the Governing Body of Jehovah’s Witnesses issued Governing Body Update #2, announced by member Gerrit Lösch, stating that members may now decide for themselves whether to have their own blood drawn, stored, and reinfused during medical care [7]. The prohibition on receiving blood from another person remains firmly in place; only PAD moved into the personal-conscience category, with the stated rationale that “the Bible does not comment on the use of a person’s own blood in medical and surgical care” [7].

This update is clinically meaningful but should not be mistaken for wholesale liberalization. As transfusion physician Caitlin Raymond has observed, the JW “conscience zone” was already substantial before this change, and individual patients vary considerably in what they accept regardless of formal doctrine [7]. Practically, PAD requires a functioning autologous donation program, specific consent (including that unused units are discarded rather than entering the general supply), and a finite donation window—typically six weeks to five days before surgery—unavailable for urgent cases. Because PAD also induces anemia through repeated phlebotomy, it complicates rather than replaces the anemia-management strategies below, and teams should continue individualized, unhurried discussion rather than assume doctrinal permission translates uniformly into patient acceptance [7].

## 3. Preoperative Anemia Management

Preoperative anemia affects 5% to 75% of surgical patients depending on population and definition, and iron-deficiency anemia (IDA) accounts for 30% to 50% of cases worldwide, with one recent analysis finding IDA present in 87% of anemic surgical patients [8]. Because preoperative anemia is the strongest predictor of perioperative transfusion, and RBC transfusion itself is an independent risk factor for adverse outcomes beyond the anemia it treats, early diagnosis and treatment are foundational to bloodless surgical care. Ideally, anemia should be identified at least 4 to 8 weeks before an elective procedure to allow time for workup (complete blood count, ferritin, transferrin saturation, and, when macrocytosis is present, vitamin B12 and folate) and treatment response [6].

For iron deficiency with lower blood-loss procedures, oral ferrous sulfate (325 mg, once a day or every other day) may suffice, though gastrointestinal intolerance and slow absorption limit its usefulness when time is short. IV iron is preferred for more severe anemia or higher blood-loss procedures; formulations vary considerably in cost (nearly 10-fold per equivalent gram), from low-molecular-weight iron dextran and ferumoxytol, capable of full repletion in a single infusion, to iron sucrose and ferric gluconate, which require smaller repeated doses [8]. The most cost-effective IV iron that we prefer for treating preoperative anemia is iron dextran (approximately $350 USD/gram, whereas other formulations can cost up to $3000 USD/gram (iron derisomaltose). Iron dextran is ideal for outpatients since a single-dose infusion (1000 mg) is often all that is necessary, avoiding the need for return visits required with iron sucrose—the other most cost effective formulation which is limited to 200 mg/dose. Earlier formulations of iron dextran (high-molecular weight) were associated with a risk of anaphylaxis, but these were taken off the market, and the newer low-molecular weight compounds have an exceedingly low risk [9]. A recent propensity-matched analysis of more than 154,000 surgical patients with preoperative IDA found that IV iron, compared with RBC transfusion, was associated with lower 30-day mortality (3.3% vs. 5.2%), lower composite morbidity (18.4% vs. 24.1%), higher postoperative Hb, and markedly reduced postoperative RBC transfusion (4.9% vs. 16.4%) [8]. These findings reinforce the physiologic rationale for bloodless anemia management: IV iron treats the cause of anemia and promotes endogenous erythropoiesis, whereas transfusion corrects only the numeric Hb value with cells bearing storage-related functional deficits.

Erythropoiesis-stimulating agents (ESAs) are reserved for more severe anemia or higher expected blood loss, typically combined with IV iron; 3 to 4 weeks of combined therapy generally raises Hb by 1 to 2 g/dL per week [10]. Because of a 2007 FDA black-box warning regarding tumor promotion and thrombotic risk, ESA use in oncology patients is generally reserved for those without curative intent, with prophylactic anticoagulation considered in the absence of active bleeding [10,11].

Our institution uses an electronic-record–derived maximum surgical blood order schedule that classifies procedures by blood-loss risk to guide an acceptable preoperative Hb target [12]. We have also described a novel Hb target formula, derived by solving the classic allowable-blood-loss equation for preoperative Hb using estimated blood volume, expected surgical blood loss, and a chosen minimum acceptable postoperative Hb (typically 8 g/dL) [13]. In a validation cohort of 25 JW spine-surgery patients, calculated target Hb values averaged 9.7 ± 0.9 g/dL, and achieved preoperative Hb exceeded this target in 92% of patients, with a lowest postoperative Hb of 6.6 g/dL and no mortality [13]. All patients should also be screened for bleeding diatheses and counseled to discontinue anticoagulants, antiplatelet agents, and bleeding-associated supplements before surgery [10,14].

The relationship between expected surgical blood loss, patient body mass, and the resulting calculated preoperative Hb target derived from this formula is illustrated in Figure 1 [13]. For example, a 100 kg patient expected to lose 1000 mL of blood during surgery needs a preoperative hemoglobin of 9 g/dL to avoid transfusion, however a 50 kg patient losing the same 1000 mL of blood needs a preoperative hemoglobin of 11 g/dL. This algorithm nicely illustrates the importance of body mass when considering management of patients who decline transfusion.

At our institution (Johns Hopkins Hospital) we have created an erythropoietic orderset for inpatients in our electronic medical record system shown in Figure 2. This is the standard therapy with standard dosing, however when patients are severely anemic (hemoglobin < 5 g/dL) we often modify therapy using higher doses discussed in Section 7. When ESAs are given, their effectiveness is enhanced with concomitant intravenous iron therapy, since iron stores are more actively utilized.

## 4. Intraoperative Blood Conservation

The central objective of intraoperative bloodless care has been summarized as “keeping the blood in the patient” [6]. Meticulous surgical technique is foundational; whatever the expected blood loss for a given operation, JW patients often lose substantially less blood than historical averages, which our surgeons attribute to heightened intentional focus on hemostasis—informally, the “Jehovah factor” [15]. Furthermore, minimally invasive approaches have also dramatically reduced bleeding: for example, robotic prostatectomy is now associated with a transfusion rate of roughly 1 in 1000 cases at our institution, compared with near-universal transfusion during open prostatectomy in the 1990s [10].

Simple physiologic measures substantially reduce bleeding. Normothermia is important because bleeding increases below 35 °C, and even mild hypothermia below 36 °C has been associated with a 16% increase in blood loss [10,16]. Controlled, mild-to-moderate hypotension (mean arterial pressure ≈70 mmHg) is particularly effective for orthopedic and spine surgery, where bone-surface bleeding cannot be controlled with sutures or cautery [10]. Antifibrinolytic agents—tranexamic acid and epsilon-aminocaproic acid—reduce blood loss and transfusion requirements by approximately 30% when all studies are considered across surgical populations [16,17]. Topical hemostatic agents and newer hemostatic electrocautery further help; a radiofrequency bipolar sealer reduced blood loss by approximately 50% in multilevel spinal fusion [10]. Point-of-care viscoelastic coagulation testing allows targeted, timely correction of coagulopathy [10].

Acute normovolemic hemodilution (ANH) is most effective with a healthy starting Hb, high anticipated blood loss, and removal of a sufficiently large volume of whole blood [10]. Blood is withdrawn into citrated bags after induction, replaced with crystalloid or colloid, and reinfused once major bleeding has stopped, restoring red cell mass along with fresh platelets and clotting factors absent from banked or salvaged blood. ANH is used most commonly in cardiac surgery [10,17]; additional cardiac-specific techniques—retrograde autologous priming, low-volume circuits, and modified ultrafiltration—further reduce hemodilution during bypass [10].

Autologous blood salvage (“cell saver”) remains one of the most valuable and, when necessary, lifesaving bloodless techniques [18]. Shed blood is suctioned into an anticoagulated reservoir, washed and concentrated by centrifugation, yielding a red cell product with a hematocrit of 55–70%. Because salvaged cells avoid prolonged storage, they retain near-normal 2,3-diphosphoglycerate and membrane deformability, unlike banked blood, suggesting more effective tissue oxygen delivery [18]. Cell salvage is generally indicated when blood loss exceeding roughly 1000 mL is expected, with approximately 57% of shed volume typically recovered [18]. The only true absolute contraindications are patient refusal and sickle cell disease (not trait) and gross bacterial contamination, while amniotic fluid contamination and cancer surgery are relative contraindications mitigated by leukoreduction filtration [18]. Multiple series in gynecologic, urologic, and spine tumor surgery using leukoreduction filters have not shown increased cancer recurrence [18]. Approximately 95% of JW patients accept cell salvage once the continuous-circuit concept is explained, often requesting that the cell saver return-line tubing remain physically connected to their IV line throughout the case [6,18].

## 5. Postoperative Management

Tolerance of lower postoperative Hb than traditionally accepted is central to bloodless postoperative care and is supported by more than a decade of restrictive-transfusion trials showing no benefit, and in several cases harm, from liberal strategies targeting 9–10 g/dL [3]. Chronic anemia is far better tolerated than acute anemia of equivalent severity; some patients tolerate Hb as low as 5 g/dL, and occasional chronically anemic patients tolerate levels as low as 3 g/dL without adverse outcome [10]. Relative decline from an individual patient’s own baseline (“delta Hb”) may be a stronger predictor of morbidity than absolute nadir Hb, and women, preconditioned by lower baseline Hb, appear to tolerate lower nadir levels than men [19].

Minimizing iatrogenic blood loss from laboratory phlebotomy is a second pillar of postoperative and inpatient bloodless management. ICU patients typically lose approximately 60 mL of blood per day to testing—more than 1% of total blood volume, roughly equal to the volume of RBCs regenerated daily through normal erythropoiesis—effectively negating erythropoietic therapy if testing is not restricted to clinically indicated draws [10]. Low-volume pediatric or neonatal microtainer tubes can reduce phlebotomy-related loss by up to 90%, and in-line reinfusion devices that minimize discard volumes reduce total ICU blood loss by 50% [10]. Many healthcare providers don’t know that phlebotomy tubes come in three general sizes—(1) adult, (2) pediatric, and (3) neonatal (Figure 3) and that the largest tubes hold 20-times the volume of blood compared to the smallest tubes. The neonatal tubes, however, are not ideal because many laboratories do not have technologies to process neonatal tubes, which must be run manually, and there is not enough left-over sample remaining to allow add-on tests, so the patient will need to be phlebotomized again if new tests are deemed necessary.

Postoperative anemia management continues with electronic order sets for vitamin B12, folate, IV iron, and EPO as indicated (Figure 2) [5]. For life-threatening anemia when blood is not an option, bovine-derived hemoglobin-based oxygen carriers such as HBOC-201 (Hemopure) and Sanguinate remain available (but very hard to obtain due to short supply), only through single-patient emergency compassionate-use protocols, given neither has completed U.S. approval [10]. Early use of HBOC-201 with Hb below 5 g/dL may improve survival, and most JW patients, once the product is explained, consider it an acceptable minor fraction, though this remains an individual decision [10].

## 6. Acceptable Blood Fractions

A patient-centered checklist approach is essential to bloodless care, since blanket assumptions about what a given JW patient will accept are frequently incorrect. Major fractions—RBCs, white blood cells, platelets, plasma, and whole blood—are unacceptable to nearly all practicing JWs and are the products a bloodless program is structured to avoid entirely. Minor fractions, including albumin, immunoglobulins, cryoprecipitate, clotting factors, prothrombin complex concentrate, and hemostatic agents, are accepted by the substantial majority once explained [6]. Techniques maintaining a continuous extracorporeal circuit with the patient’s own blood—cell salvage, ANH, cardiopulmonary bypass, hemodialysis, and epidural blood patch—fall into the same personal-conscience category and are accepted by most patients [6].

The doctrinal landscape shifted meaningfully with the March 2026 Governing Body Update permitting PAD as a personal-conscience decision [7]. For bloodless programs, this creates a new, narrow pathway: a JW patient referred early enough for elective surgery at an institution with an autologous donation program may now bank units of their own blood for possible reinfusion, in addition to—not instead of—the intraoperative and postoperative techniques above. This does not apply to urgent surgery, to institutions lacking autologous collection capacity, or to the majority of JW patients worldwide without access to well-resourced centers [7]. Because PAD itself can worsen preoperative anemia through repeated phlebotomy, any patient pursuing it should undergo the same anemia workup described earlier, and unused units are discarded rather than entering the general blood supply—a detail worth disclosing during consent [7]. Without iron and/or erythropoietin, preoperative autologous donation may not be effective in reducing transfusion since there is often not enough time between donation and surgery to regenerate red blood cells.

Consistent with our long-standing approach to minor fractions and personal-choice techniques, all patients pursuing bloodless care should complete an individualized checklist identifying which products and procedures they will and will not accept, entered into the electronic record and updatable at any time [6]. This should be paired with an honest, unhurried conversation led by a bloodless coordinator—at many centers themselves a JW congregation member—since formal doctrinal permission does not automatically translate into individual acceptance given the social and religious context of such decisions [6,7].

## 7. Managing Non-Surgical Patients Who Don’t Accept Transfusion

Although bloodless medicine is often discussed in a surgical context, over half of patients cared for by our program are medical inpatients: in our largest reported cohort of 1111 adult bloodless patients, 62.8% were medical rather than surgical, most commonly admitted for sickle cell crisis, pneumonia, myocardial infarction, heart failure, chronic obstructive pulmonary disease, renal failure, cardiac arrhythmia, chest pain, or septicemia. The same core principles apply: minimizing iatrogenic phlebotomy loss, aggressively treating anemia with oral or IV iron and, when appropriate, EPO, vitamin B12, or folate, and tolerating lower Hb than conventionally targeted [5]. A consultant hematologist and bloodless-program anesthesiologist are available for every patient, supported by nurse coordinators—often JW congregation members themselves—who discuss blood products in the context of religious doctrine and help resolve family disagreement at the bedside [5,6].

Pediatric patients present a distinct challenge, since U.S. courts have consistently held, beginning with the 1944 Supreme Court decision in Prince v. Massachusetts, that parents cannot legally withhold lifesaving treatment from their children on religious grounds [20,21]. At Johns Hopkins we use the following approach. Families are told directly that every effort will be made to avoid transfusion, and we often cite the methods we use from Table 1 which reassures the parents, but we also tell them that providers are legally obligated to transfuse if a child’s life or health is threatened. It is challenging, however, to determine what severity of anemia defines this threshold. In our largest pediatric series (196 patients), only 6.1% ultimately received an allogeneic blood component—most commonly for perioperative bleeding or anemia of prematurity—and none required a court order [21]. Pediatric bloodless patients had lower composite morbidity than standard-care controls (2.6% vs. 6.2%) and no mortality, with similar length of stay and costs [21]. Constant, transparent family communication allows most transfusion decisions to occur proactively, before a true emergency, substantially reducing (or avoiding) confrontational court proceedings [21]. In our 13 years of operating a bloodless program we have never needed a court order using this approach.

A remarkable example of how well patients can do with severe anemia is illustrated in a publication by Sklar, et al. [22] Two patients with gastrointestinal (GI) bleeding were admitted to our facility with a nadir hemoglobin of 2.1 g/dL and 2.8 g/dL, respectively. Using what we call the “super-high-dose” erythropoietic therapy, along with other treatment modalities (Table 2), both of these patients left the hospital alive and well. These are, however, anecdotal cases using off-label rescue strategies, without a proven risk to benefit ratio supporting safety.

## 8. Clinical Outcome Studies and Cost-Effectiveness

The strongest evidence for bloodless medicine comes from propensity-matched, risk-adjusted comparisons at high-volume centers. In an early 294-patient cohort matched 1:4 to standard-care controls, bloodless care was not an independent predictor of the composite adverse outcome after risk adjustment (odds ratio [OR] 1.02), while in-hospital mortality was, unexpectedly, lower in the bloodless group (0.7% vs. 2.7%), and total and direct hospital costs were 12% and 18% lower. An expanded analysis of 1111 adult bloodless patients compared with 137,009 standard-care patients found similar composite morbidity (14.4% vs. 16.0%) and length of stay; hospital-acquired infection occurred significantly less often in bloodless medical (but not surgical) patients (4.3% vs. 8.3%; adjusted OR 0.56), and median total hospital costs were 8.7% lower overall [5]. A plausible mechanism for the infection finding is the immunomodulatory effect of allogeneic transfusion, supported by a 2014 meta-analysis of 18 trials showing an 18% relative reduction in hospital-acquired infection with restrictive transfusion strategies [5].

Figure 4 illustrates the individual morbid event rates for the overall bloodless compared to standard care cohort, with hospital-acquired infection showing the largest, and only statistically significant, difference between groups [5].

Pediatric outcomes follow a similar pattern: among 196 bloodless children compared with 37,271 standard-care controls, composite morbidity was lower in the bloodless cohort (2.6% vs. 6.2%), with no deaths and no significant difference in hospital charges or costs [21]. Cardiac surgery literature from multiple centers has generally reported equivalent or, in some analyses, better outcomes—including lower rates of myocardial infarction and reoperation for bleeding—among bloodless patients compared with matched controls [23,24].

Financial analyses reinforce these clinical findings. A recent 10-year evaluation of our combined health-system PBM program and Center for Bloodless Medicine and Surgery found the PBM clinical community reduced annual blood acquisition costs by $2.9 million (9.6-fold return on investment), while the bloodless program generated approximately $5 million per year in variable net margin (6.6-fold return), for a combined program-wide return on investment of 7.5-fold—more than seven dollars saved or generated per dollar invested—with outcomes unchanged or improved and hospital-acquired infection significantly lower among bloodless patients [25]. Comparable IV iron versus RBC transfusion data in the broader surgical population further support the premise that reducing reliance on allogeneic transfusion can simultaneously improve outcomes and reduce cost [8].

These findings warrant caveats. All are observational and subject to selection bias: patients who decline transfusion may be denied elective surgery if perceived risk is unacceptable without transfusion as a backup, potentially selecting a healthier bloodless surgical cohort, and populations are heterogeneous with respect to diagnosis and procedure [5]. Nonetheless, the consistency of these findings across adult, pediatric, medical, and surgical subgroups over more than a decade at a single high-volume center supports that a well-structured bloodless program delivers care that is ethically, clinically, and financially sound. The generalizability of patient care and clinical outcomes for other centers with less experience in bloodless medicine may be in question, since optimizing care for these patients involves a learning curve.

## 9. Conclusions

Caring for patients who decline allogeneic blood transfusion—most commonly members of the Jehovah’s Witness faith—requires a structured, multidisciplinary approach spanning the preoperative, intraoperative, and postoperative periods: early diagnosis and treatment of anemia, meticulous attention to intraoperative hemostasis and blood conservation technology such as cell salvage and ANH, and postoperative tolerance of lower Hb thresholds combined with minimized iatrogenic blood loss. When delivered by an experienced team at an established center, this approach may yield clinical outcomes equivalent to or better than standard transfusion-inclusive care, at lower cost, in adult, pediatric, medical, and surgical populations alike. Because these techniques were originally developed for the roughly 1% of patients who decline transfusion but demonstrably benefit outcomes and cost more broadly, bloodless medicine is increasingly understood not as a niche accommodation but as a template for high-value PBM applicable to all patients.

The doctrinal landscape continues to evolve, as illustrated by the March 2026 Governing Body Update permitting preoperative autologous donation as a matter of personal conscience. This change expands, but does not simplify, the clinical conversation: it adds a narrow new option for appropriately selected, well-resourced, electively scheduled patients, while leaving the core prohibition on allogeneic transfusion—and the wide individual variability in what patients will accept—unchanged. Clinicians should maintain updated fluency in doctrine, offer individualized, unhurried, private discussion of all options, and continue relying on the checklist-based, multidisciplinary framework proven safe and effective over more than a decade of published outcomes. Continued prospective research and broader institutional investment in bloodless infrastructure will help extend these benefits to the growing number of patients who wish to avoid allogeneic transfusion.

## Figures and Tables

**Figure 1:**
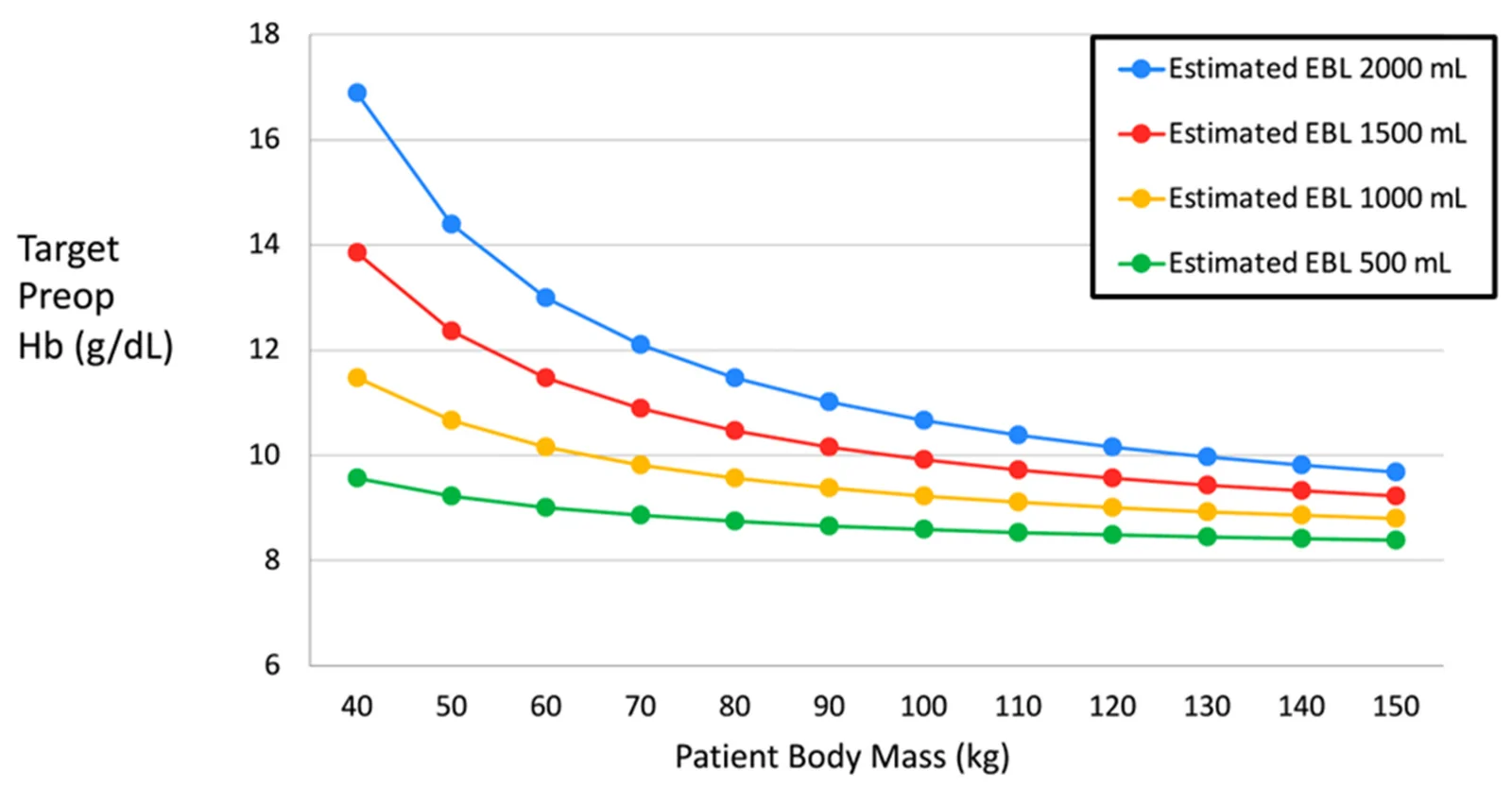
Nomogram demonstrating the ideal target preoperative hemoglobin (Hb) based on varying estimated blood loss (EBL) and patient body mass, with a postoperative allowable Hb of 8 g/dL, calculated using the preoperative Hb target formula. Hb—hemoglobin, EBL—expected blood loss. Adapted with permission from Ref. [13] Copyright © 2023 Elsevier.

**Figure 2:**
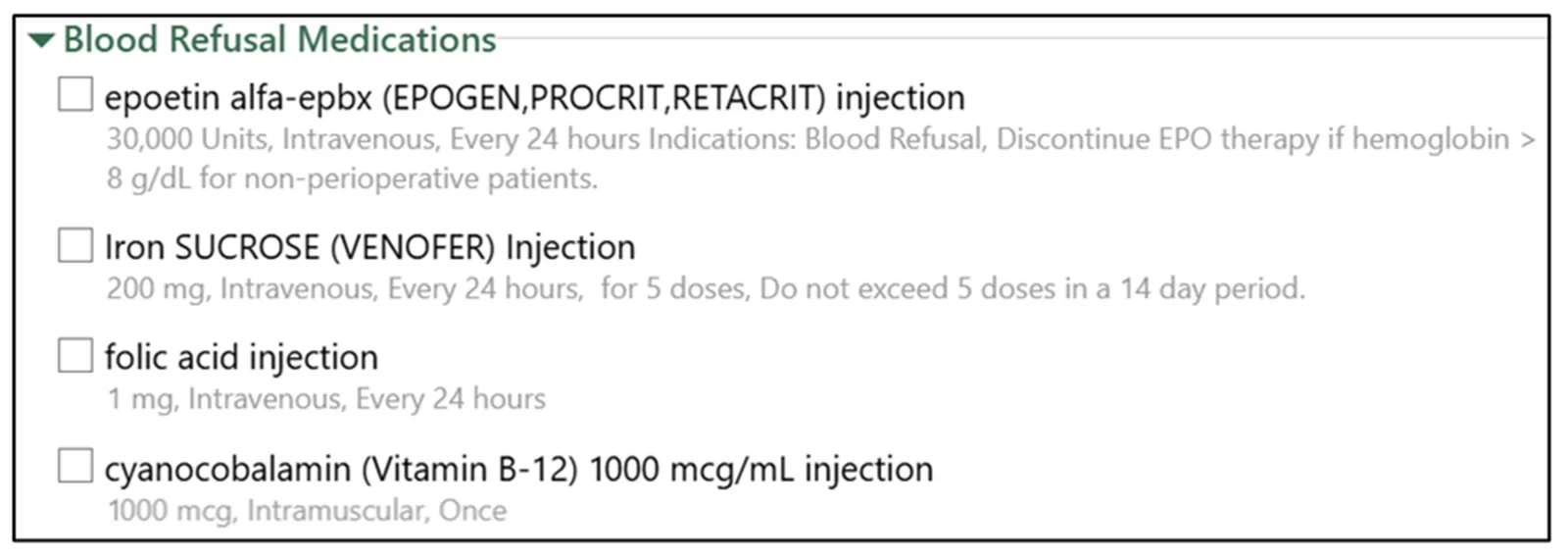
Standard erythropoietic orderset used at Johns Hopkins Hospital for treating anemia for inpatients who decline transfusion. This orderset includes doses, frequency, routes of administration, and duration of therapy.

**Figure 3:**
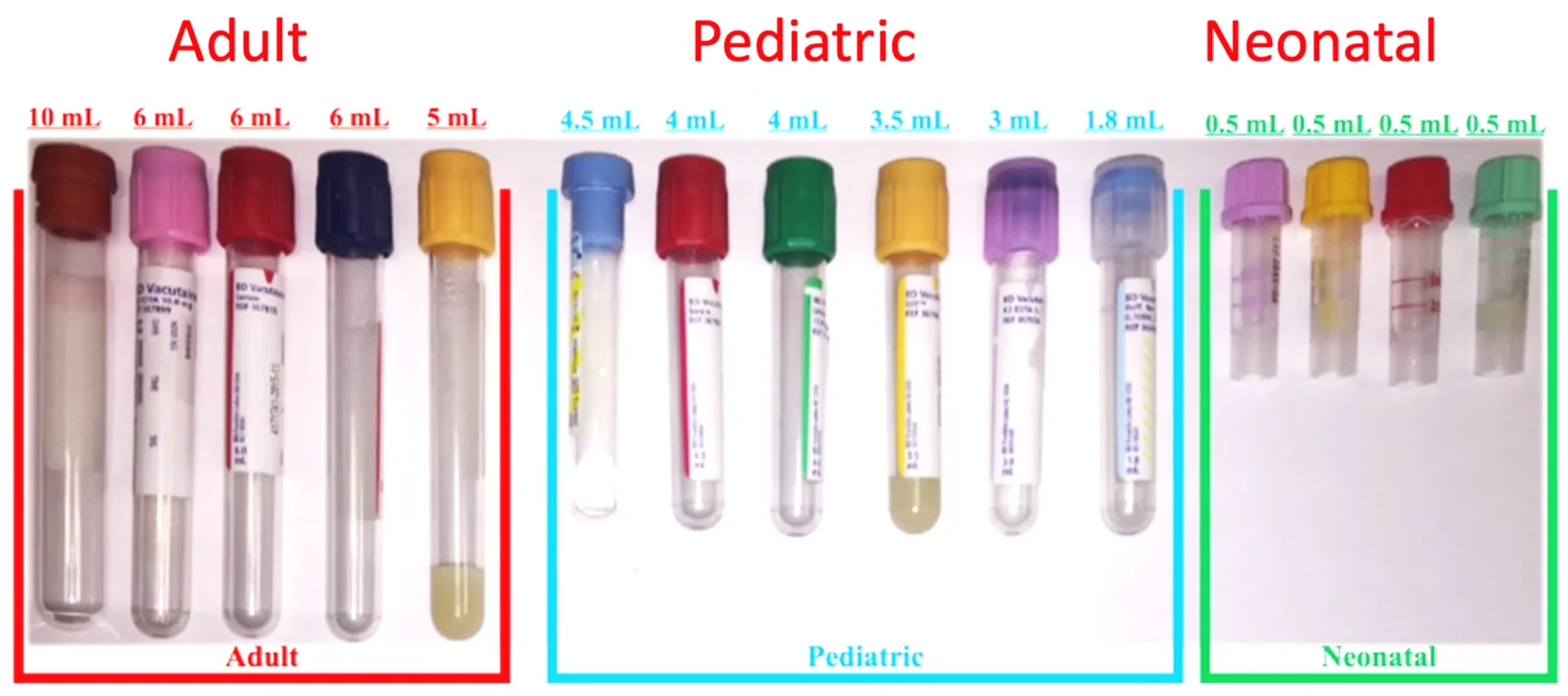
The three categories of sizes for phlebotomy tubes. Note that the largest tubes hold 10 mL and the smallest 0.5 mL.

**Figure 4:**
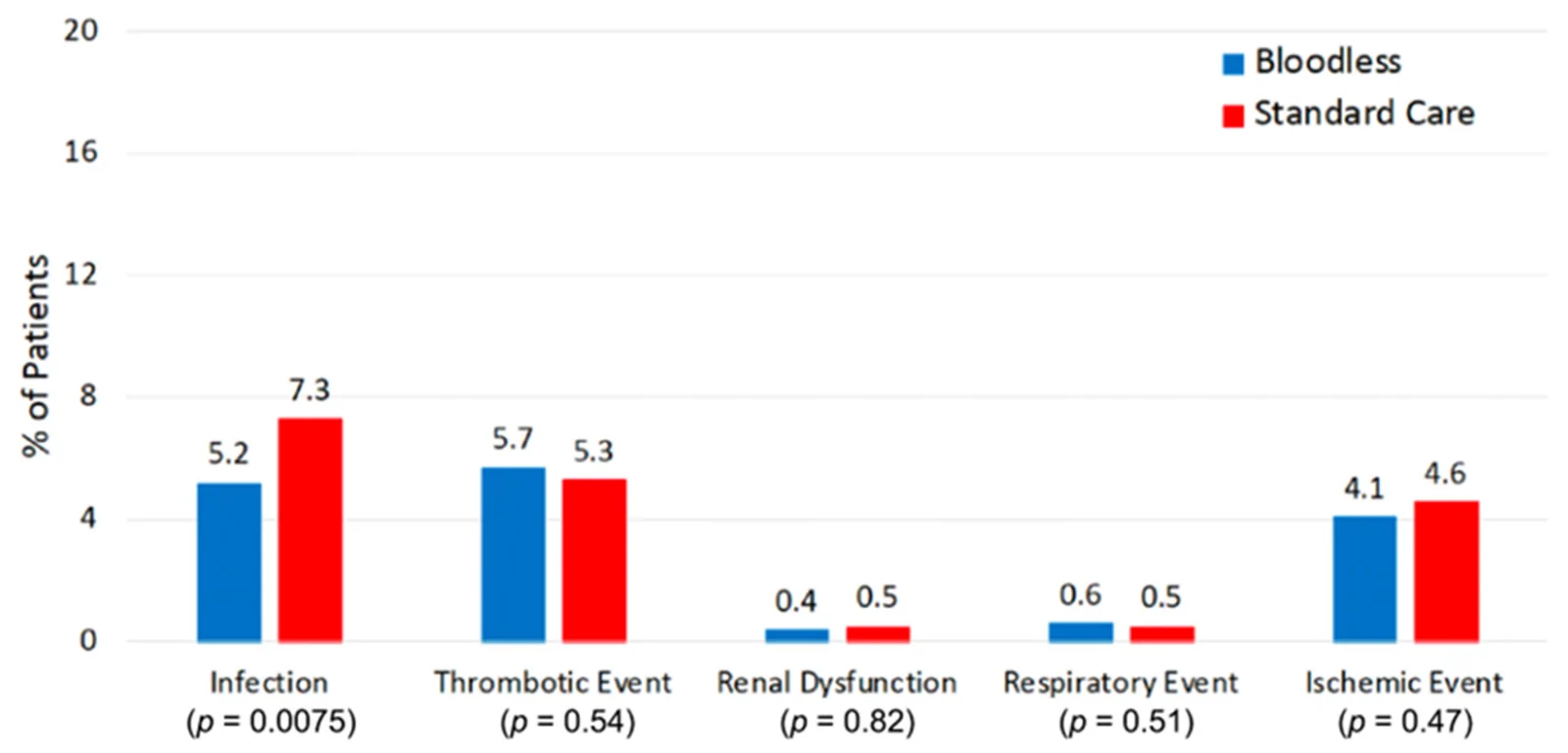
Individual morbid events for adult bloodless and standard care patients, all patients. Adapted with permission from Ref. [5] Copyright © 2022 International Anesthesia Research Society.

**Table 1:** Bloodless Medicine Techniques.

	Methods
Preoperative	Diagnosis and treatment of preoperative anemia
	Management of iron deficiency
	Discontinuing anticoagulants appropriately
Intraoperative	Meticulous surgical technique
	Minimally invasive surgical techniques (e.g., robotic)
	Maintaining perioperative normothermia
	Intraoperative autologous cell salvage
	Intraoperative acute normovolemic hemodilution
	Controlled hypotension
	Topical hemostatic agents
	Antifibrinolytic drugs (eg. tranexamic acid)
	Point-of-care coagulation testing (viscoelastic testing)
Postoperative	Decreasing the frequency of blood draws
	Utilizing pediatric-sized phlebotomy tubes
	Tolerating lower hemoglobin levels
	In-line blood return devices for arterial and central venous catheters

Adapted with permission from Ref. [6] Copyright © 2021 International Anesthesia Research Society.

**Table 2:** Methods Used to Manage Severe Anemia (hemoglobin < 3 g/dL) in Two GI Bleeding Patients.

GI endoscopy to identify and cauterize bleeding
Nuclear scan with tagged red blood cells to identify bleeding site
Interventional radiology—attempted embolization to stop GI bleeding
Tranexamic acid 1 g intravenous loading dose
Tranexamic acid 3 mg/kg/h intravenous infusion
Epoetin alpha 40,000 units intravenous every 12 h
Darbepoetin 300 units intravenous or subcutaneous once (or twice) per week
Iron sucrose 300 mg intravenous every day
Folate 1 mg orally every day
Vitamin B12 1000 µg orally every day
Minimize phlebotomy—fewer blood draws and use of pediatric tubes (0.5 mL capacity)

Abbreviation: GI, gastrointestinal. Adapted with permission from Ref. [22] Copyright © 2024 International Anesthesia Research Society.

## Data Availability

Data supporting reported results can be found by contacting S.M.F. at sfrank3@jhmi.edu. Databases searched for relevant literature (from 1977–2026): Pubmed/Medline, Embase, Web of Science, Scopus.

## Abbreviations

The following abbreviations are used in this manuscript:

AABB	Association for Advancement of Blood and Biotherapeutics
ABT	Allogeneic blood transfusion
ANH	Acute normovolemic hemodilution
EBL	Expected blood loss
ESA(s)	Erythropoiesis-stimulating agent(s)
GI	Gastrointestinal
Hb	Hemoglobin
HBOC	Hemoglobin-based oxygen carrier
IDA	Iron-deficiency anemia
JW	Jehovah’s Witness
OR	Odds ratio
PAD	Preoperative autologous donation
PBM	Patient blood management
TRICC	Transfusion Requirements in Critical Care
